# Unexplained deaths due to possibly infectious causes in the United States: defining the problem and designing surveillance and laboratory approaches. The Unexplained Deaths Working Group.

**DOI:** 10.3201/eid0201.960106

**Published:** 1996

**Authors:** B A Perkins, J M Flood, R Danila, R C Holman, A L Reingold, L A Klug, M Virata, P R Cieslak, S R Zaki, R W Pinner, R F Khabbaz

**Affiliations:** National Center for Infectious Diseases, Centers for Disease Control and Prevention, Atlanta, Georgia, USA.


### Unexplained Deaths Due to Possibly Infectious Causes in the United States: Defining the Problem and Designing Surveillance and Laboratory Approaches

Many new infectious diseases have been identified in the United States during the last several decades (1). Among these are AIDS, Legionnaires' disease, toxic-shock syndrome, hepatitis C, and most recently, hantavirus pulmonary syndrome; all caused serious illness and death. In each instance, the disease was recognized through investigation of illness for which no cause had been identified. Retrospective studies of these and other newly recognized infectious diseases often identified cases that occurred before the recognition of the new agent; therefore, a more sensitive detection system may make the earlier recognition of new infectious agents possible.

Delays in recognizing new infectious agents have often been substantial. For instance, *Legionella pneumophila* was established as the cause of Legionnaires' disease in 1976 after an epidemic in Philadelphia, but sporadic cases in 1947 and an outbreak in 1957 were retrospectively identified (2, 3). Similarly, toxic shock syndrome was recognized in late 1979 and early 1980, but retrospective reporting and chart reviews documented cases as early as 1960 (4). HIV was identified in 1983 (5) yet retrospective investigations documented AIDS cases in the late 1970s and possibly as early as 1968 in the United States (6, 7).

The difficulty of identifying unknown etiologic agents is part of the reason for delays between the occurrence and recognition of new infectious diseases. Until recently, to identify new infectious agents we relied primarily on culture techniques. For fastidious bacteria such as *Legionella* sp., and new viruses, such as HIV, which have very specific growth requirements, successful isolation usually required numerous attempts with various culture systems, often extending over years. Advances in molecular techniques, including polymerase chain reaction (PCR) amplification and other DNA- (and RNA-) based techniques (e.g., representational difference analysis), allow identification and classification of unknown etiologic agents without having to culture them (8-10) and provide clues concerning appropriate conditions for subsequent isolation of the agent in culture (11,12).

A more systematic public health approach for the early detection of unknown infectious agents is needed. This need was acknowledged in *Addressing Emerging Infectious Diseases Threats: A Prevention Strategy for the United States*, a CDC publication about emerging infections (13). CDC has established an emerging infections program (EIP) network to conduct special population-based surveillance projects, develop surveillance methods, pilot and evaluate prevention strategies, and conduct other epidemiologic and laboratory studies. In late 1994, CDC funded four programs based at state health departments and academic institutions in California (Alameda, Contra Costa, Kern, and San Francisco counties), Connecticut, Minnesota, and Oregon. Some projects are conducted at all program sites and others, depending on local interest and expertise, at only one or two sites.

Surveillance for unexplained deaths due to possibly infectious causes (UDPIC) for early detection of new infectious diseases is one of the core activities being conducted at all sites. This paper estimates the number of UDPIC at the EIP programs and summarizes the surveillance and laboratory approaches that will be used to identify their cause. This is the first attempt to conduct surveillance for early detection of new infectious diseases in a large U.S. population.

To estimate the number of deaths that might be identified in surveillance for UDPIC, we used multiple cause-of-death data for the United States for 1992 from the National Center for Health Statistics (14). The year 1992 was the most recent for which national data were available at the time of this study. The analyses of death records were restricted to the EIP program populations and age group (1-49 years of age) in which surveillance for UDPIC was planned. Multiple cause-of-death data listed on the National Center for Health Statistics death record allow for analysis of mortality data based on the different causes (15). The International Classification of Diseases, 9th Revision (ICD-9) was used to define UDPIC (16). We selected 77 codes likely to represent UDPIC when listed on the death record (Table 1) (17).

Table 1. Selected codes from International Classification of Diseases, 9th revision (ICD-9) used to identify unexplained deaths due to possibly infectious causes (UDPIC)

| | |
|---|---|
| 007.9 | unspecified protozoal intestinal disease |
| 008.5 | bacterial enteritis, unspecified |
| 008.8 | intestinal infectious due to other organisms: other organism, not classified elsewhere |
| 009.0 | infectious colitis, enteritis, and gastroenteritis |
| 009.1 | colitis, enteritis, and gastroenteritis of presumed infectious origin |
| 009.2 | infectious diarrhea |
| 009.3 | diarrhea of presumed infectious origin |
| 027.9 | unspecified zoonotic bacterial disease |
| 038.9 | unspecified septicemia |
| 041.9 | bacterial infection in conditions classified elsewhere and of unspecified site: bacterial infection, unspecified |
| 046.9 | unspecified slow virus infection of the central nervous system |
| 047.9 | unspecified viral meningitis |
| 049.9 | unspecified non-arthropod-borne viral diseases of central nervous system |
| 057.9 | viral exanthem, unspecified |
| 079.9 | viral infection in conditions classified elsewhere and of unspecified site: unspecified viral and chlamydial infection |
| 099.0 | venereal disease, unspecified |
| 136.9 | other and unspecified infectious and parasitic diseases: unspecified infectious and parasitic diseases |
| 283.1 | non-autoimmune hemolytic anemias |
| 284.8 | other specified aplastic anemias |
| 286.6 | defibrination syndrome |
| 287.3 | primary thrombocytopenia |
| 287.5 | thrombocytopenia, unspecified |
| 320.9 | meningitis due to unspecified bacterium |
| 322.9 | meningitis, unspecified |
| 323.9 | unspecified cause of encephalitis |
| 357.0 | acute infective polyneuritis |
| 420.9 | other and unspecified acute pericarditis |
| 421.0 | acute and subacute bacterial endocarditis |
| 421.9 | acute endocarditis, unspecified |
| 422.9 | other and unspecified myocarditis |
| 424.9 | endocarditis, valve unspecified |
| 425.4 | other primary cardiomyopathies |
| 425.9 | secondary cardiomyopathy, unspecified |
| 446.6 | thrombotic microangiopathy |
| 465.0 | acute laryngopharyngitis |
| 465.8 | acute upper respiratory infections of multiple or unspecified sites: other multiple sites |
| 465.9 | acute upper respiratory infections of multiple or unspecified sites: unspecified site |
| 466.0 | acute bronchitis |
| 466.1 | acute bronchiolitis |
| 480.9 | viral pneumonia, unspecified |
| 482.9 | bacterial pneumonia, unspecified |
| 485 | bronchopneumonia, organism unspecified |
| 486 | pneumonia, organism unspecified |
| 511.9 | unspecified pleural effusion |
| 518.4 | acute edema of lung, unspecified |
| 518.8 | other diseases of lung |
| 519.9 | unspecified disease of respiratory system |
| 558 | other and unspecified noninfectious gastroenteritis and colitis |
| 780.6 | pyrexia of unknown origin |
| 782.1 | rash and other nonspecific skin eruption |
| 782.7 | spontaneous ecchymoses |
| 785.5 | shock without mention of trauma |
| 785.6 | enlargement of lymph nodes |
| 786.0 | dyspnea and respiratory abnormalities |
| 792 | nonspecific abnormal findings in other body substances |
| 792.0 | cerebrospinal fluid |
| 792.1 | stool contents |
| 792.2 | semen |
| 792.3 | amniotic fluid |
| 792.4 | saliva |
| 792.9 | other nonspecific abnormal findings in body substances |
| 795 | nonspecific abnormal histologic and immunologic findings |
| 795.3 | nonspecific positive culture findings |
| 795.4 | other nonspecific abnormal histologic findings |
| 795.7 | other nonspecific immunologic findings |
| 796.4 | other nonspecific abnormal findings: other abnormal clinical findings |
| 798 | sudden death, cause unknown |
| 798.1 | instantaneous death |
| 798.2 | death occurring in less than 24 hours from the onset of symptoms, not otherwise explained |
| 798.9 | unattended death |
| 799 | other ill-defined and unknown causes of morbidity and mortality |
| 799.0 | asphyxia |
| 799.1 | respiratory failure |
| 799.3 | debility, unspecified |
| 799.4 | cachexia |
| 799.8 | other ill-defined conditions |
| 799.9 | other unknown and unspecified cause |

Analyses for UDPIC were restricted to previously healthy persons 1 to 49 years of age by excluding persons outside this age-group and those who had any of the following ICD-9 codes as an underlying cause of death: 140 to 239.9, neoplasms; 250.0 to 250.9, diabetes mellitus; 279.0 to

279.9, disorders involving the immune mechanism; 295.5, other disease of spleen; 800 to 999.9, injury and poisoning; E800 to E998, supplementary classification of external causes of injury and poisoning. Patients with HIV disease listed anywhere on the death record were also excluded (codes 042, 042.0, 042.1, 042.2, 042.9, 043, 043.0, 043.1, 043.2, 043.3, 043.9, 044, 044.0, 044.9, and 795.8) (18).

Deaths meeting the study criteria were identified along with patient age, gender, race (black, white, and other), and autopsy status for the four EIPs (aggregate and by EIP program). To determine rates of UDPIC, we used 1992 census estimates for the four EIP programs (19).

In 1992, 744 UDPIC were identified among previously healthy persons 1 to 49 years of age in the four EIP sites. These deaths accounted for 14% of all deaths (n = 5,304) among persons 1 to 49 years of age in hospitals and emergency rooms. Most of the 744 UDPIC occurred among male patients (60%) and whites (72%) (Table 2). Overall rates among blacks were almost four times as high as those among whites (29.5 vs. 7.7 per 100,000). By site, overall rates ranged from 5.6 (in Minnesota) to 14.5 (in California) per 100,000 population. These geographic differences could be accounted for only in part by differences in the proportions of blacks by site. In Minnesota and Oregon the proportions of blacks were 2.8% and 1.9%, respectively, whereas in California and Connecticut the proportions were 14.7% and 12.4%, respectively.

Figure 1 shows the age-specific rates of UDPIC for persons 1 to 49 years of age. Persons 1 to 24 years of age accounted for only 19% of deaths, while persons 40 to 49 years of age accounted for 50%.

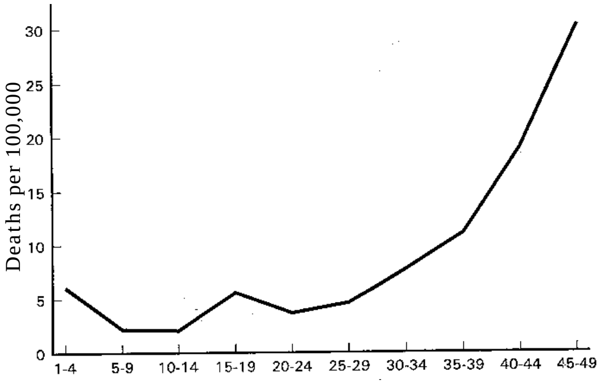

Figure 1. Age-specific rates of unexplained deaths due to possibly infectious causes (UDPIC) among previously healthy persons 1 to 49 years of age in the four emerging infections program sites, 1992.

Of selected ICD-9 codes (Table 1), the six disease classifications (and codes) accounting for the most of the UDPIC are shown in Table 3. A selected ICD-9 code was listed as the underlying cause of death in 253 (34%) of 744 UDPIC. Autopsies were performed in 293 (39%) of the 744 UDPIC.

Two approaches for surveillance were proposed as a basis for the EIP project. In the first, clinicians will be asked to report unexplained deaths and serious illnesses from possibly infectious causes. In the second, death certificate databases will be used to select patients with ICD-9 codes likely to represent UDPIC. The first approach allows prospective collection of data and specimens for deaths and serious illnesses. In the second approach, UDPIC will be identified retrospectively through information on death certificates.

Clinicians in the EIP areas have been asked to report by telephone to EIP program surveillance personnel all previously healthy persons 1 to 49 years of age who are hospitalized (or admitted to

Table 2. Unexplained deaths due to possibly infectious causes (UDPIC) among previously healthy persons by emerging infection program (EIP) site, 1992

| | | Rate (per 100,000 population aged 1-49 years) | | | | | |
|---|---|---|---|---|---|---|---|
| | | | Gender | | Race | | |
| EIP site | No. of UDPIC | Overall | Female | Male | Black | White | Other |
| California* | 316 | 14.5 | 10.8 | 18.5 | 34.0 | 12.2 | 8.9 |
| Connecticut† | 83 | 14.2 | 10.5 | 18.5 | 37.9 | 11.4 | - |
| Minnesota | 189 | 5.6 | 4.8 | 6.6 | 11.0 | 5.4 | 9.5 |
| Oregon | 156 | 7.2 | 6.9 | 7.7 | 21.8 | 7.0 | 7.8 |
| Total | 744 | 8.9 | 7.4 | 10.9 | 29.5 | 7.7 | 8.7 |

*Alameda, Contra Costa, and San Francisco counties.
†New Haven County.

Table 3. Of selected ICD-9 codes, disease classifications accounting for most unexplained deaths due to possibly infectious causes (UDPIC) in the four study sites, 1992

| | UDPIC with ICD-9 code included on death record by age group (%) | | | |
|---|---|---|---|---|
| Disease classification (ICD-9)* | 1–49 yr; n = 744 | 1–14 yr; n = 75 | 15–39 yr; n = 295 | 40–49 yr; n = 374 |
| Respiratory failure (799.1) | 205 (28) | 14 (19) | 91 (31) | 100 (27) |
| Unspecified septicemia (038.9) | 108 (14) | 8 (11) | 42 (14) | 58 (16) |
| Pneumonia, organism unspecified (486) | 101 (14) | 7 (9) | 33 (11) | 61 (16) |
| Other primary cardiomyopathy (425.4) | 84 (11) | 5 (7) | 26 (9) | 53 (14) |
| Shock without mention of trauma (785.5) | 83 (11) | 10 (13) | 29 (10) | 44 (12) |
| Other unknown or unspecified (799.9) | 75 (10) | 9 (12) | 35 (12) | 31 (8) |
| Totals† | 505 (68) | 39 (52) | 193 (65) | 273 (73) |

*More than one of these disease classifications (ICD-9 code) may be listed on a death record.
†UDPIC with at least one of the six disease classifications included on the death record.

an emergency room) with a life-threatening illness with hallmarks of an infectious disease for which no cause is identified. Inclusion and exclusion criteria are shown below.

**Inclusion criteria**

1. 1 to 49 years of age
2. Admitted to a hospital or emergency room with life-threatening illness of potentially infectious etiology
3. No cause for illness identified by preliminary testing

**Exclusion criteria**

1. Preexisting chronic medical condition: malignancy; HIV infection; chronic cardiac, pulmonary, renal, hepatic or rheumatologic disease; or other known underlying chronic illness (e.g., diabetes mellitus)
2. Immunosuppressive therapy
3. Trauma
4. Toxic ingestion or exposure
5. Nosocomial infection

Clinicians and pathologists in the four EIP programs were informed of the surveillance system through a combination of mailings, oral presentations, and posters.

Classifying patients as having one or more infectious disease-related syndrome(s) as listed below should help identify groups of patients with similar illnesses for laboratory testing.

1. Acute abdominal symptoms (e.g, diarrhea, pain, nausea/vomiting) and history of (h/o) fever
2. Arthritis or osteomyelitis and h/o fever
3. Blood cell dyscrasia or coagulopathy and h/o fever
4. Conjunctivitis, keratitis, endophthalmitis, or periocular infection and h/o fever
5. Endocarditis, myocarditis, pericarditis and h/o fever
6. Hepatitis or hepatic insufficiency/failure and h/o fever
7. Meningitis, encephalitis, encephalopathy, dementia, or other neurologic syndrome with or without a h/o of fever
8. Rash, skin or mucosal membrane lesions, cellulitis, myositis, lymphadenitis, or lymphangitis and h/o of fever
9. Renal insufficiency/failure and h/o of fever
10. Respiratory failure, pulmonary infiltrates, or other pleuropulmonary manifestation and h/o of fever
11. Shock or sepsis and h/o of fever or hypothermia
12. Other

Information about exposures (e.g., travel or contact with animals or insects) resulting in infectious diseases will be collected. For patients who are still alive or have died recently, clinical and pathology laboratories will be asked to save clinical specimens (including biopsied tissues) obtained during clinical care and diagnostic evaluation. Range of specimens will vary but be appropriate for the given illness and organ systems affected. These specimens will be collected, divided into aliquots, and stored. Autopsies will be encouraged. With the exception of pathology specimens, specimens will be initially banked at the EIP sites. Fixed or frozen tissue specimens (premortem and postmortem) will be sent directly to CDC for examination. A CDC pathologist will be available to consult with the local pathologist and to discuss preparation and transport of tissues. Pathology results are expected to guide further laboratory testing on specimens.

Clinical and epidemiologic data will be periodically reviewed locally at each EIP and at CDC in aggregate. Each EIP will identify UDPIC not reported through the clinician-based system by using state-based (rather than national) electronic data systems to reduce delays in relaying information. When deaths not reported through the clinician-based system are identified, the medical chart will be reviewed, the patient's illness will be classified by syndrome and information available in the medical record concerning exposures will be collected. Samples of specimens will be obtained at autopsy. Deaths will be handled as in the clinician-based system with regard to periodic review and laboratory testing, although it is expected that fewer clinical specimens will be available from patients whose deaths were not reported through the clinician-based system.

Additional reference level laboratory tests for known pathogens will be done in state health laboratories and CDC. CDC will test for previously unrecognized infectious agents.

Initial identification of unrecognized etiologic agents at CDC will primarily rely on serology, immunohistochemistry, and nucleic acid probes. When a sufficient number of patients with similar illnesses are identified, a customized strategy for laboratory testing will be designed. Serology and immunohistochemistry will be used to narrow the scope of possible etiologies. Nucleic acid probes will be used with PCR to amplify from clinical specimens specific fragments of genetic material that can be sequenced and used for phylogenetic comparisons to known infectious agents. Clinicians who reported cases will be informed of laboratory results, but information will usually not be available in time to affect treatment of individual patients.

Until now, unexplained deaths and serious illnesses due to possibly infectious causes have not been addressed as a specific public health problem. The data obtained in the first phase of this project suggest that UDPIC in previously healthy persons account for 13% of hospitalized deaths among persons 1 to 49 years old in the EIP sites. Experience in recent years with new infectious diseases suggests that systematic study of UDPIC and similarly unexplained serious illnesses may allow earlier detection of emerging infections. This has been made more feasible by newly developed nucleic acid-based methods for identification of unknown etiologic agents.

Use of the 1992 National Center for Health Statistics multiple cause-of-death data to estimate the number of UDPIC has its limitations. The most important is in the selection of ICD-9 codes to identify these deaths. Even with codes such as 038.9 ("unspecified septicemia"), which seem relevant, without reviewing the medical record it is impossible to know if the cause of the septicemia was known by the clinician but not specified or was nosocomial. Codes representing potentially infectious deaths (e.g., 799 for "other ill-defined and unknown causes of morbidity and mortality") might also be assigned to noninfectious deaths. Another critical limitation is failure to identify deaths that are, in fact, unexplained but have been given an incorrect diagnosis.

For several reasons, our surveillance is limited to persons 1 to 49 years of age who have been healthy. The 1-year lower age limit was selected to avoid confusion with congenital problems in infants but include most children in day-care, where infectious diseases are common and a new infectious disease might spread rapidly. The upper age limit was set to exclude an expected increased proportion of unexplained deaths from noninfectious causes in persons 50 years and older. Many of the recently recognized life-threatening infectious diseases would have been detected among previously healthy persons in this age-group. Previously healthy persons might also be considered better sentinels for new infectious diseases because of their generally more vigorous interaction with people and higher likelihood of exposure to infections (e.g., travel or contact with animals or insects). However, restricting surveillance to previously healthy persons is likely to decrease the sensitivity of our system.

Patients who are immunocompromised—whether from HIV infection, malignancy, or immunosuppressive therapy—and many patients with other chronic illnesses, are more susceptible to known and unknown infectious diseases. New infectious diseases first identified in persons who are immunocompromised or have chronic illnesses have subsequently been found to also cause infection in persons with normal immune systems (20,21). Although sensitivity could be improved by including these populations in surveillance, available resources and a concern that laboratory evaluation would be complicated by the broader range of infectious possibilities compelled us to focus on previously healthy persons.

Clinician-based and death certificate–based systems for surveillance and laboratory evaluation are being used in combination because of their complementary strengths and weaknesses. The notable strengths of the clinician-based system are the contribution of clinicians and the timeliness of reporting. Because of their training and their relationship with patients, clinicians can recognize unusual and potentially new infections. This system also offers opportunities to collect and store clinical specimens (pre-mortem and post-mortem) that would not normally be saved, in addition to providing systematic and timely collection of exposure information that might not be available in the medical record. This system might also increase the likelihood of an autopsy. However, reporting is time-consuming and is not likely to affect the patient's care, which may lower the sensitivity of this approach.

The primary strengths of the death certificate–based system are its completeness and relative ease, once the data are electronically available. The completeness may make it sensitive for detection of new infections resulting in death (but assumes that the correct ICD-9 codes are selected and that they are coded accurately). Sensitivity is important because, to be effective, the combined approaches should detect relatively rare illnesses (e.g., in the range of one case per 100,000 to 1,000,000 population per year). The main disadvantages of this system are the vagaries of ICD-9 classification: codes are not designed to identify new infectious diseases and are assigned by persons not directly familiar with the case. The list of ICD-9 codes used to identify UDPIC is likely to be modified on the basis of information collected in this system and in the clinician-based system. Another problem is the delay in getting information on the death certificate into the database for review, which makes this system relatively slow. Further, the only clinical specimens likely to be available for laboratory evaluation are those collected at autopsy.

The goal of our project is early detection of new life-threatening infectious diseases. However, it is likely that in the process, we will identify cases in which known, but poorly recognized, infectious diseases are responsible, either because the diagnostic tests being used clinically are of poor sensitivity or because the diagnosis was unexpected by clinicians. Findings concerning such cases may be useful in identifying areas in which better diagnostic capabilities are needed and in improving estimates of infectious disease prevalence (22). A population-based bank of clinical specimens will be invaluable in current and future testing for newly recognized etiologic agents and for developing diagnostic tests. This project will better clarify surveillance strategies and help standardize nucleic acid-based techniques for identification of previously unknown etiologic agents. Through it, we expect to build U.S. capacity for detecting and responding to newly recognized infectious diseases not only at the EIP sites but elsewhere, nationally and internationally.

**Bradley A. Perkins,* Jennifer M. Flood,† Richard Danila,‡ Robert C. Holman,* Arthur L. Reingold, Laura A. Klug,* Michael Virata,§ Paul R. Cieslak,¶ Sherif R. Zaki,* Robert W. Pinner,* Rima F. Khabbaz,* and the Unexplained Deaths Working Group#**

*National Center for Infectious Diseases, Centers for Disease Control and Prevention, USA, Atlanta, Georgia; †School of Public Health, University of California at Berkeley, California, USA; ‡Minnesota Department of Health, Minneapolis, Minnesota, USA; §Yale University School of Medicine, New Haven, Connecticut, USA; ¶Oregon Department of Human Resources, Portland, Oregon, USA

#The Unexplained Deaths Working Group: Grechen Rothrock, University of California at Berkeley; Duc Vugia, California Department of Health Services; James Hadler, Matt Cartter, Connecticut Department of Public Health and Addiction Services; James Meek, Robin Ryder, Mark Wilson, Yale University School of Medicine; Michael Osterholm, Kristine L. MacDonald, Jean Rainbow, Norman Crouch, Kathy LeDell, Minnesota Department of Health; David Fleming, Katrina Hedberg, Oregon Health Division; Don Brenner, Mark Eberhard, James Olson, Pierre Rollin, R. Gibson Parrish, CDC.

### References

1. Institute of Medicine. Emerging infections: microbial threats to health in the United States. Washington, DC: National Academy Press, 1992.
2. McDade JE, Brenner DJ, Bozeman FM. Legionnaires' disease bacterium isolated in 1947. Ann Intern Med 1979;90:659-61.
3. Osterholm MT, Chin TDY, Osborne DO, et al. A 1957 outbreak of Legionnaires' disease associated with a meat packing plant. Am J Epidemiol 1983;117:60-7.
4. Osterholm MT, Forfang JC. Toxic-shock syndrome in Minnesota: results of an active-passive surveillance system. J Infect Dis 1982;145:458-64.
5. Barre-Sinoussi F, Chermann JC, Rey F, et al. Isolation of a T-lymphotropic retrovirus from a patient at risk for acquired immunodeficiency syndrome (AIDS). Science 1983;220:868-71.

6. CDC Task Force on Kaposi's Sarcoma and Opportunistic Infections. Epidemiologic aspects of the current outbreak of Kaposi's sarcoma and opportunistic infection. N Engl J Med 1982;306:248-52.
7. Garry RF, Witte MH, Gottlieb A, et al. Documentation of an AIDS virus infection in the United States in 1968. JAMA 1988;260:2085-7.
8. Relman DA, Loutit JS, Schmidt TM, Falkow S, Tompkins LS. The agent of bacillary angiomatosis: an approach to the identification of uncultured pathogens. N Engl J Med 1990;323:1573-80.
9. Nichol ST, Spiropoulou CF, Morzunov S, et al. Genetic identification of a hantavirus associated with an outbreak of acute respiratory illness. Science 1993; 262:914-7.
10. Chang Y, Cesarman E, Pessin MS, et al. Identification of herpesvirus-like DNA sequences in AIDS-associated Kaposi's sarcoma. Science 1994;266:1865-9.
11. Koehler JE, Quinn FD, Berger TG, LeBoit PE, Tappero JW. Isolation of *Rochalimaea* species from cutaneous and osseous lesions of bacillary angiomatosis. N Engl J Med 1992;327:1625-31.
12. Elliot LH, Ksiazek TG, Rollin PE, et al. Isolation of the causative agent of hantavirus pulmonary syndrome. Am J Trop Med Hyg 1994;51:102-8.
13. Centers for Disease Control and Prevention. Addressing emerging infectious disease threats: a prevention strategy for the United States. Atlanta: U.S. Department of Health and Human Services, Public Health Service, 1994.
14. National Center for Health Statistics. Public use data tape documentation. Multiple cause of death for ICD-9 1992 data. Hyattsville, MD: U.S. Department of Health and Human Services, 1994.
15. Israel RA, Rosenberg HM, Curtin LR. Analytical potential for multiple cause-of-death data. Am J Epidemiol 1986;124:161-79.
16. World Health Organization. Manual of the international statistical classification of diseases, injuries, and causes of death, based on the recommendations of the Ninth Revision Conference, 1975, and adopted by the twenty-ninth World Health Assembly. Vol. 1. Geneva: World Health Organization, 1977.
17. Chamblee RF, Evans MC. TRANSAX, the NCHS system for producing multiple cause-of-death statistics, 1968-78. Washington, D.C.: US Government Printing Office, 1986. DHHS Pub No. [PHS] 86-1322).
18. National Center for Health Statistics. Vital statistics of the United States 1987, Vol. II, mortality, part A, technical appendix. Washington, D.C.: U.S. Department of Health and Human Services, Public Health Service, 1990. (DHHS Pub No. (PHS) 90-1101).
19. U.S. Bureau of Census. Intercensal estimates of the population of counties by age, sex and race: 1970-1992 (machine-readable data file). Washington, DC: U.S. Bureau of Census, 1995.
20. MacKenzie WR, Hoxie NJ, Proctor ME, et al. A massive outbreak in Milwaukee of *Cryptosporidium* infection transmitted through the public water supply. N Engl J Med 1994;331:161-7.
21. Tappero JW, Koehler JE, Berger TG, et al. Bacillary angiomatosis and bacillary splenitis in immunocompetent adults. Ann Intern Med 1993;118:363-5.
22. Pinner RW, Teutsch S, Simonsen L, et al. Trends in infectious diseases mortality in the United States. JAMA 1996;275:189-93.
